# Mobile device: a useful tool to teach inhaler devices to healthcare professionals

**DOI:** 10.1186/s12909-022-03302-0

**Published:** 2022-04-02

**Authors:** Ser Hon Puah, Chee Yen Goh, Chung Leung Chan, Amy Kui Jie Teoh, Hao Zhang, Zhiqi Shen, Lay Ping Neo

**Affiliations:** 1 Respiratory and Critical Care Medicine Department, Tan Tock Seng Hospital, 11 Jalan Tan Tock Seng, Singapore, 308433 Singapore; 2 Department of Nursing, Tan Tock Seng Hospital, Singapore, Singapore; 3 LILY (Joint NTU-UBC Research Centre of Excellence in Active Living for the Elderly), Nanyang Technological University, Singapore, Singapore; 4 School of Computer Science and Engineering, Nanyang Technological University, Singapore, Singapore

**Keywords:** Asthma, Inhaler, Mobile application, Inhaler device

## Abstract

**Background:**

Proper inhaler device usage is paramount for control of underlying obstructive airway disease. Hence, education to healthcare professionals who will eventually educate patients need to be done effectively. We developed an application for mobile devices for education on six medical inhaler devices, the metered-dose inhaler (MDI), Turbuhaler, Accuhaler, Breezhaler, Ellipta and Respimat, and studied if there were any difference between the application and the manufacturer’s instructions on inhaler technique. The aim of this study is to see if inhaler education via a mobile phone app is comparable to manual instruction for health care professions.

**Methods:**

Participants, who were nursing students, were randomized to learn the inhaler devices via the manufacturer’s instruction guide or a mobile device app designed specifically for education on inhaler devices.

**Results:**

There were 45 participants in each group. 78% of them were females with a median age of 21 (IQR 3). 67% used an Apple mobile device and the remainder used an Android device. The mobile device showed better total improvement points for the Turbuhaler device (262 vs 287 points; *P* = 0.02). Participants learning from the manufacturer’s guide had a significantly higher total improvement points in the Breezhaler (370 vs 327 points; *P* < 0.01) and Ellipta (214 vs 174 points; P < 0.01) device. Both interventions showed improvement in total scores for demonstrating the correct usage of all inhaler devices. MDI has the least number of correct steps for both interventions. The participants’ reported their mean (SD) self-rated knowledge was significantly higher for those using the app for all devices as compared to those that did not (4.33 (0.68) vs 4.73 (0.42); P = < 0.01). Self-reported confidence level was found to be higher in the mobile app group, but this was not statistically significant. The app was well received and scored of 4.42 of 5 with regards to its quality.

**Conclusion:**

Using a mobile inhaler app is just as effective to teach inhaler device techniques to healthcare professionals and is likely a more convenient, versatile and important adjunct to learning.

**Trial registration:**

National Healthcare Group Ethics Board (2018/00960).

**Supplementary Information:**

The online version contains supplementary material available at 10.1186/s12909-022-03302-0.

## Background

Inhaled bronchodilators and corticosteroids are crucial to the control of chronic obstructive airway diseases such as bronchial asthma and chronic obstructive pulmonary disease (COPD). Guidelines have stressed the importance of proper selection and education to patients to ensure effective delivery of inhaled medications [1, 2]. Recommendations include training patients in the proper usage of inhalers and regular assessment of inhaler techniques [3]. Poorly performed inhaler techniques leads to poor outcomes [4, 5]. Quality and effective instructions given by health care professionals (HCP) are key to decrease inhaler mishandling [6].

Previous studies have shown that only 7–28% of HCPs were able to demonstrate the correct metered-dose inhaler (MDI) technique [7–9]. A person with suboptimal skills will not be able to educate patients and ensure correct and proper handling of their device. Therefore, it is equally important for HCPs to be equipped with the proper skills and knowledge before they start training patients. In one study, only 13–15% of house staff were able to demonstrate perfect inhaler technique in a traditional large group lectures and handouts [10]. More effective and efficient ways to train HCPs are needed before they take on the task of training patients [11].

Medical devices and software applications (App) s are gradually integrating and assimilating into the medical education [12, 13]. Whether it is on a laptop, portable mobile tablets or personal mobile phones, it is now seen as an important adjunct to learning [14]. Mobile apps are convenient as it allows learning to happen at any time, any place and it is easily accessible [15]. Mobile devices enhance learner engagement and provide instant means of assessment and feedback [16, 17]. App development and mobile technology is advancing rapidly, and it is important that it should remain relevant and the contents appropriate for the target audience. As information is now being digitized, we need to be able to learn to adapt and find innovative ways to direct learning through electronic means.

The current practice for educators and practitioners who are not familiar with the inhalers or have forgotten how to use these devices will have to rely on instructions provided by the company along with recollection of previous tutorials which may not be recent. It has been the main form of reference for educators and HCP responsible for dispensing medications as the instructions are included in the medication package. Reading from the manufacturer’s instructions alone or even watching videos on how to use the inhalers have been shown to be inferior to having a guide [18, 19]. Having been thought and shown how to use the devices is insufficient for retention in healthcare students. Initial learning only happens during the school period and over time, this knowledge will need to be reenforced or it will dissipate [20]. Having an easily accessible personal tutor would likely address these shortfalls in retention of knowledge.

To date, there is no smartphone application available for HCPs to acquire inhaler technique. We developed a mobile phone app to create a reliable resource for HCPs and students to learn inhaler technique and be a source of reference at any time to facilitate just-in-time learning. The app allows the use of finger gestures and phone movements to simulate an inhaler. Using Kolb’s learning cycle of reflective observation by looking at graphic and video demonstrations, conceptualisation of the mechanisms of the different devices, allowing active repeated experiementation to allow the formation of concrete experiences [21]. We hope to evaluate this new method of teaching using an electronic device compared to traditional manual instructions.

We aim to evaluate the effectiveness of learning inhaler skills through a mobile app by assessing acquisition of knowledge and if users are able to demonstrate proper inhaler techniques. We also assessed whether the app is user friendly and to see how receptive users are to learning inhaler techniques through an app.

## Methods

### Design

**Fig. 1:**
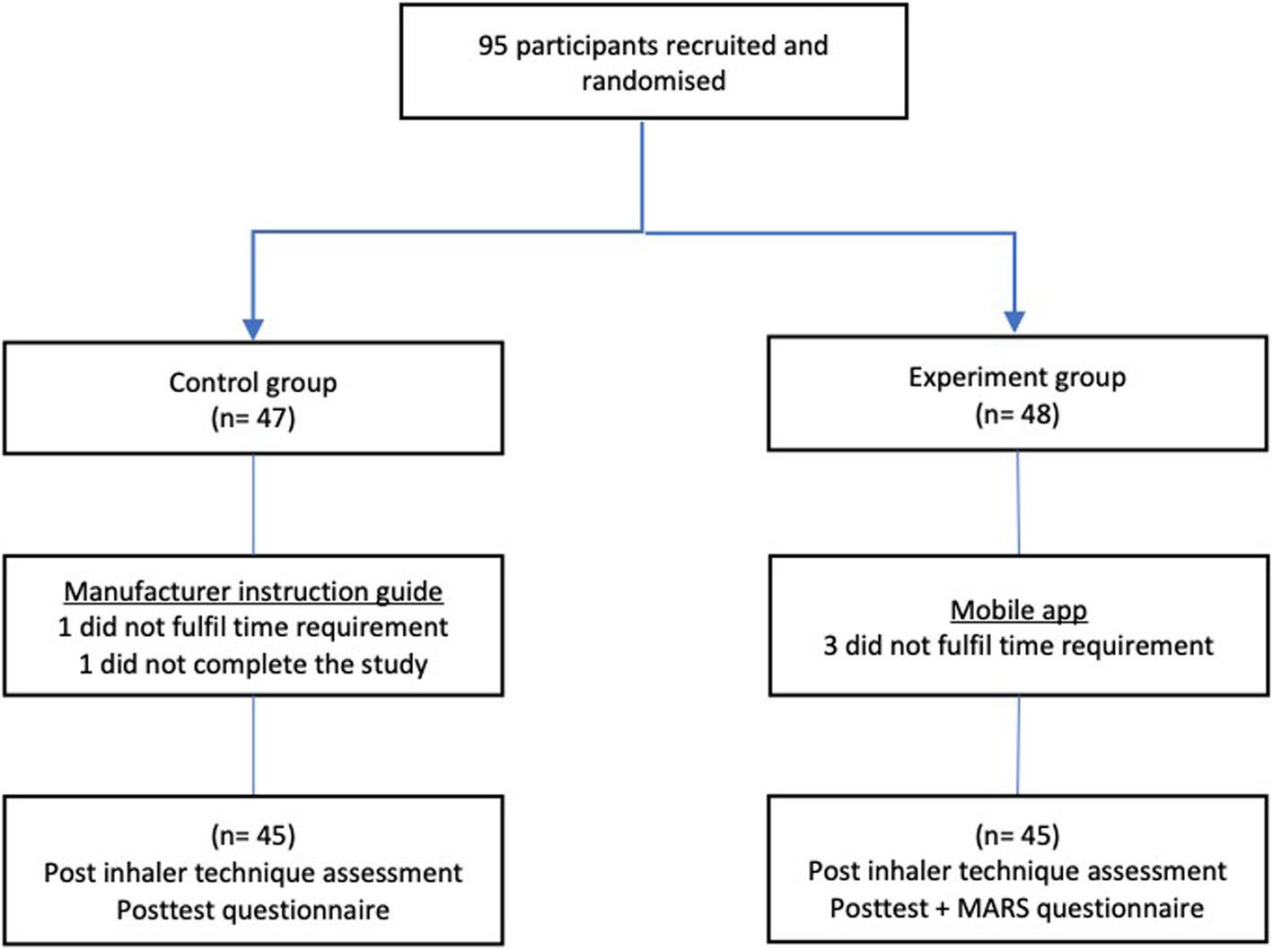
Randomization process during recruitment of participants

This was a randomised controlled trial using a pre and post-test design. The study was conducted from 3rd January to 28th March 2019 in Tan Tock Seng Hospital and was approved by the National Healthcare Group Ethics Board (2018/00960). Participants who were randomised into the control group received the manufacturer’s hard copy instruction guide for all devices that were studied to learn the inhaler technique. Participants who were randomized in the experimental group were given the mobile application Fig. 1. A research assistant was onsite for both groups to help with randomisation and data collection. No direct instructions on inhaler techniques were given to the participants by the research assistant. A medical statistician generated the randomisation numbers via a randomisation software. The randomised results were sealed in individual envelops.

### Participants

Participants were final year nursing students on their Pre-registration Clinical Placement (PRCP) clinical attachment. The inclusion criteria were (1) Nursing students (2) > 18 years old (3) willing to participate in the study. Participants who (1) had previously worked as an enrolled nurse, (2) had upper respiratory tract infection such as cough, running nose, fever and sore throat, (3) has asthma or any other chronic respiratory condition, (4) or suffering from any medical condition which precludes them from participating, will be excluded from the study. Consent was taken prior to the start of the study. All participants who completed the study received a SGD10 gift card.

### App development

A mobile application was developed in English from June 2017 to December 2017 for the Android platform. It engaged learners through three learning styles (i.e., visual, auditory and kinaesthetic), providing for a multisensory approach to learning without the need for physical inhaler devices and human supervision.

**Fig. 2:**
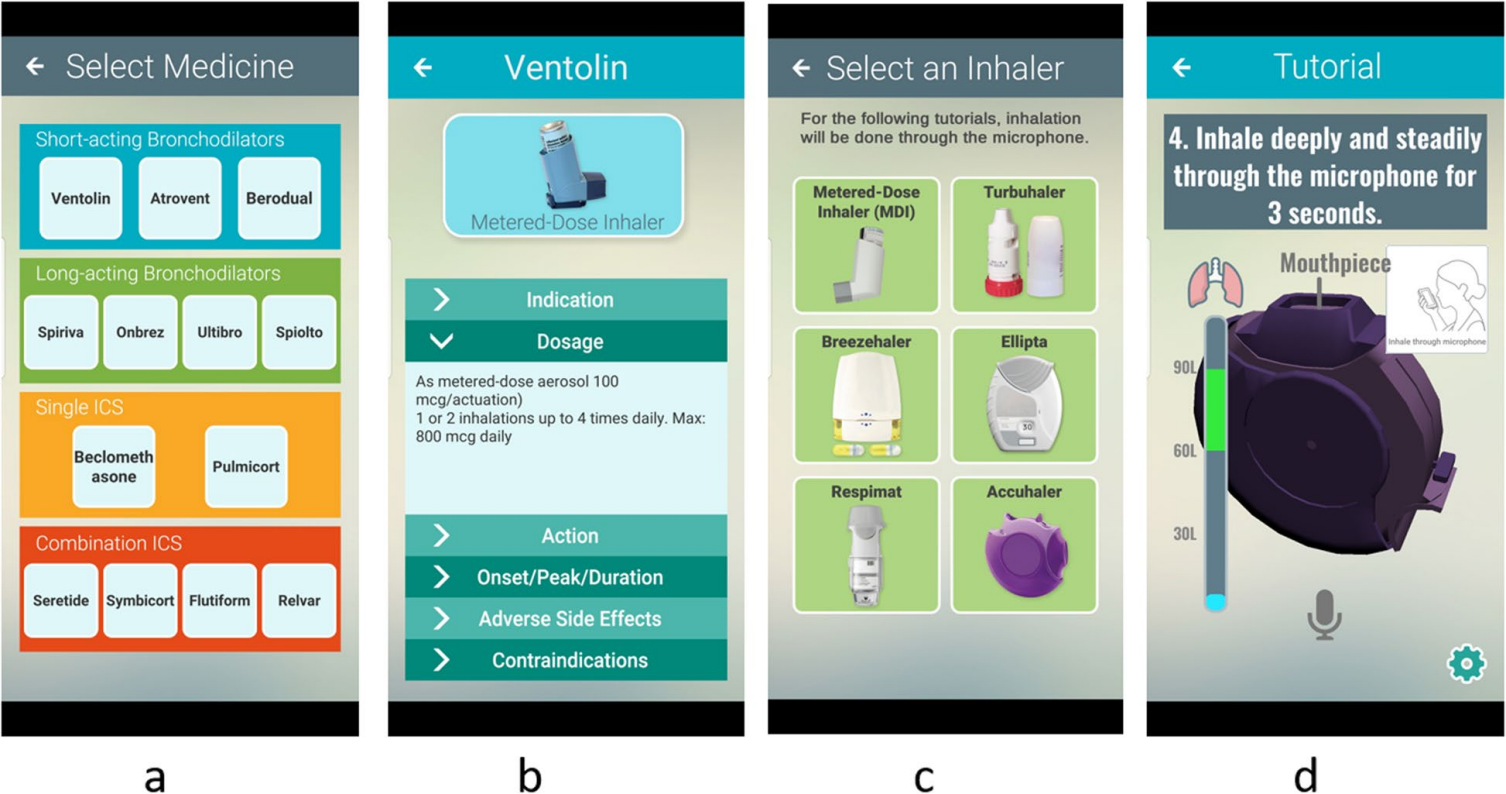
Mobile app interface

The app recreated accurate 3-dimensional models of different types of inhaler devices and remapped steps of using an inhaler onto a mobile phone device. The design was to ensure an intuitive and interaction experience to learn the handling of inhalers. These were achieved through touch manipulation, hand motion and respiratory detection. The app detects touch and swipe gestures near the different parts of the 3D inhaler models on the screen. The user can swipe on the canister of the MDI inhaler to depress it. White smoke particles will emit through the mouth of inhaler in response. The app also uses the mobile phone’s built-in microphone to detect a range of low frequency sounds, specifically the sound produced by inhaling, within a consistent period, and reacts to the detection. Inhaling while white smoke emits from the MDI inhaler model will make particles move faster, representing flow and to give the users a visual sense that they are inhaling the smoke. The Breezhaler model will produce a rattling sound while inhaling, mimicking the medicine tab rattling within the inhaler’s chamber in real life. Finally, the app uses the phone’s accelerometer to detect vertical shake action. The MDI inhaler model will respond to the shake and shake by itself on screen. The purpose is to allow learners to freely manipulate inhalers while learning the correct inhaler technical skills. Screenshots and features of the app are shown in Fig. 2.

To ensure correctness, visual guides tell learners how to manipulate inhalers step by step. These were reinforced by visual and audio cues, such as leaking of gas at improper inhaling, providing positive reinforcements and constructive corrections. A demonstration feature was included to give an option to run through steps on how to use an inhaler. The feature resides before the interactive inhaler tutorials, providing students a choice to learn by observing before attempting themselves.

The student assessment quiz was included in the app to assess students of their knowledge to inhalers and related medications. The quiz contained multiple choice questions, which allowed immediate evaluation and conveyed the appropriate answer back to the student. At the end of the quiz, results were displayed to be recorded by assessors.

### Intervention

The study consisted of a pre and post-test inhaler technique assessment and a post-intervention questionnaire at the end of the study. Six inhaler devices were used for this study i.e. metered-dose inhaler, Turbuhaler, Accuhaler, Breezhaler, Ellipta and Respimat as these are the common inhalers prescribed in the public hospitals and clinics in Singapore. An assessment checklist compiled from the manufacturers’ instructions brochures was used for the pre and post-test inhaler assessment Additional file 1. The assessors were 3 trained respiratory nursing clinicians with more than 5 years of experience in the field of respiratory medicine from the institution. Each inhaler step is counted as 1 point and the total points for each inhaler recorded before and after each intervention. A pre-test assessment was conducted and each participant were subsequently allocated a minimum of 30 mins up to 90 min to learn all 6 inhaler devices.

### Post-test survey

All participants had to complete a questionnaire at the end of the study. The questionnaire was divided into five sections: demographic, prior experience with using mobile phones, prior experience on inhaler devices, self-reported confidence in using the inhaler and self-rated knowledge level. Participants will mark on a scale of not at all, not very, somewhat, very and completely for the self-reported confidence level, while the self-rated knowledge level was marked on a scale between strongly disagree, disagree, neutral, agree and strongly agree.

In addition to the post-test survey, the experiment group were asked questions pertaining to the app. App related questions were adopted from the Mobile app rating scale (MARs) questionnaire to evaluate the app quality, functionality and graphic design [22]. It consisted of questions asking users on their perception of the app’s engagement, functionality, aesthetics and information quality using a Likert scale from 1 to 5 (1 strongly disagree and 5 strongly agree). The participants were also asked on the subjective quality of the app.

### Statistical analysis

Estimating the mean inhaler technique knowledge level in the control group to be 20% and the mean inhaler technique knowledge level in the inhaler mobile app group to be 50% so that the knowledge levels difference to be normally distributed with a standard deviation of 0.5, we will need to study 90 subjects in total, to be able to reject the null hypothesis that there is no difference in mean inhaler technique knowledge level between control and experimental group, with power of 80% and Type I error probability of 0.05. Continuous variables were expressed as mean (standard deviation; SD) or median (interquartile range; IQR) depending on distribution, and categorical variables were expressed as frequency and percentage. We compared differences for continuous variables using two-sample *t* test depending on the distribution, and χ^2^ test or Fisher’s exact test for categorical variables. χ^2^ and Fisher exact tests were applied to evaluate the effectiveness of the App on learning inhalers compared with control group. The data analysis is conducted with respect to all steps for each module as well as to each step for each module. Mann-Whitney U test was used to analyze participants’ confidence level and knowledge between the two groups. Analyses was done using Stata (version 13.1, College Station, TX: StataCorp LP).

## Results

A total of 95 participants were recruited and randomized for the study. One from the control group did not complete the study while 1 from the control group and 3 from the mobile app group spent less than 30 min on the app and hard copy instruction guides and were subsequently excluded. 45 participants from each group eventually completed the study and were subsequently analysed. 78% of participants were female and the median age group was 21 (IQR 3) Baseline characteristics of the participants are shown in Table 1. In brief, there were no significant differences between the control and experiment groups in terms of gender distribution, age, and ethnicity. 66.7% of the participants were on an Apple based mobile device while 33% used an Android based mobile device. 33 (73%) in the control group and 34 (76%) in the mobile app group had no prior personal experience using any inhalers. 21 (47%) and 20 (44%) in the control group and the mobile app group respectively had no prior teaching experience with regards to inhaler technique and use.

**Table 1:** Baseline characteristics of enrolled participants

	All (*n* = 90)	Control group (*n* = 45)	Mobile app group (*n* = 45)	*P* value
**Demographics**				
Female	70 (78)	33 (73%)	37 (82%)	0.31
Age, median (IQR)	21 (3)	21 (5)	21 (3)	0.90
**Ethnicity**				
Chinese	67 (74%)	36 (80%)	31 (69%)	0.11
Malay	9 (10%)	6 (13%)	3 (7%)	
Indian	6 (7%)	1 (2%)	5 (11%)	
Others	8 (9%)	2 (5%)	6 (13%)	
**Mobile model**				
Apple	60 (67%)	28 (62%)	32 (71%)	0.37
Android	30 (33%)	17 (38%)	13 (29%)	
**Prior experiences**				
No experience using any inhalers	67 (74%)	33 (73%)	34 (76%)	0.64
No experience teaching any inhaler	41 (46%)	21 (47%)	20 (44%)	0.45

For both groups, all participants had a significant improvement in all inhaler techniques Table 2. The mobile app group was found to have done better with the Turbuhaler device after the intervention. (319 vs 298 points; *P* = 0.02). The control group did better with the Breezhaler and Ellipta device after the intervention. (370 vs 327 points; P = 0.02 and 214 vs 174 points; P = < 0.01 respectively) There were no difference with regards to the correct usage of the other inhaler devices when using the mobile app compared to conventional teaching of inhalers. Post intervention, both groups achieved the least number of correct steps in MDI device Table 3.

**Table 2:** Improvement in inhaler demonstration before and after interventions between both groups

Device	Control group		*P* value	Mobile app group		*P* value
Steps correctly demonstrated	Pre intervention	Post-intervention		Pre-intervention	Post-intervention	
Accuhaler (Total points, 315)	63 (20.0%)	290 (92.1%)	*p <* 0.01	89 (28.3%)	280 (88.9%)	*p <* 0.01
Turbuhaler (Total points, 360)	36 (10.0%)	298 (82.8%)	*p <* 0.01	32 (8.9%)	319 (88.6%)	*p <* 0.01
Respimat (Total points, 315)	35 (11.1%)	263 (83.5%)	*p <* 0.01	24 (7.6%)	265 (84.1%)	*p <* 0.01
Breezhaler (Total points, 450)	47 (10.4%)	417 (92.7%)	*p <* 0.01	56 (12.4%)	383 (85.1%)	*p <* 0.01
Ellipta (Total points, 270)	39 (14.4%)	253 (93.7%)	*p <* 0.01	62 (23.0%)	236 (87.4%)	*p <* 0.01
MDI (Total points, 315)	140 (44.4%)	264 (83.8%)	*p <* 0.01	138 (43.8%)	264 (83.8%)	*p <* 0.01

**Table 3:** Total point improvement for each inhaler device post intervention

Device	Control group	Mobile app group	*P* value
Accuhaler	227	191	0.067
Turbuhaler	262	287	0.02
Respimat	228	241	0.665
Breezhaler	370	327	< 0.01
Ellipta	214	174	< 0.01
MDI	124	126	0.946

all scores are calculated based on improvement in total steps done correctly after intervention

The participants’ reported their mean (SD) self-rated knowledge was significantly higher for those using the app for all devices as compared to those that did not (4.33 (0.68) vs 4.73 (0.42); P = < 0.01). Although self-reported confidence level was found to be higher in the mobile app group, however, it was not statistically significant Table 4.

**Table 4:** Self-rated knowledge and confidence level

	Control group	Mobile app group	z	*P* value
Knowledge level	4.33 (0.68)	4.73 (0.42)	−3.056	< 0.01
Confidence level (overall)	3.95 (0.75)	3.98 (0.73)	−0.184	0.85

Ninety-eight percent of users of the mobile app felt that it was a useful teaching tool. All users of the app found it to be engaging (4.24 of 5), functional (4.47 of 5), aesthetically pleasing (4.44 of 5) and informative (4.42 of 5). Subjectively, participants would recommend this to people who they thought would benefit from it (4.51). Participants felt that they will use the app about 3–10 times in the next 12 months. The app was given an overall score of 3.98 out of 5. Despite the well-received performance of the app, the participants were less likely to pay money to purchase the app (score of 2.2 of 5) Table 5.

**Table 5:** App quality and app subjective rating

	Score
**App quality (mean score 4.42)**	
Information	4.52
Aesthetics	4.44
Functionality	4.47
Engagement	4.24
**App subjective quality rating (mean 3.53)**	
Would you pay for the app	2.2
How many times would you use the app in the next 12 months	3.42
Would you recommend this app	4.51
Overall rating	3.98

## Discussion

The education of proper use of inhaler devices especially among student nurses, who may potentially teach patients the proper administration of this life saving device, is of paramount importance. To the knowledge of the authors, this is the first ever reported randomized controlled trial using a mobile app to teach inhaler devices. This study provided opportunities for learners to acquire inhaler techniques and knowledge in a non-classroom setting using a mobile device which is now ubiquitous to everyone. This was compared with the manufacturer’s guide which comes standard in the medication’s packaging and are used as the main form of reference for educators and HCP responsible for dispensing medication to patients. We showed that learning via a mobile app did improve the knowledge and confidence of users. Although this finding was also seen in the group using hard copy learning materials, it was non inferior. There were increase in self-reported knowledge after both interventions. This finding is in keeping with previous studies which revealed an increased in self-reported knowledge after using the app. In one study, 71% of the participants experienced a self-reported improvement in antibiotic knowledge after accessing the application [23].

Higher learning have been shown to improve based on instructional design features such as interactivity, practice exercises, repetition, and feedback [24]. Users use experiential learning to construct knowledge and improve on their technique based on the visual cues and the easily accessible information customized within the app itself [21]. The development of this mobile app was based on a multisensory approach. It allowed intuitive interaction through touch manipulation, respiratory detection, audio and visual cues for immediate feedback. Hence, it is evident that the intuitive interactive features of the app had a significant impact in acquiring inhaler technique skills. This study was done comparing the outcomes on a single time point and immediately post intervention, which may explain why the results in both arms were somewhat comparable. Future studies will have to be done to see if knowledge is retained after a period of time and how convenient it is for HCP to seek reference or practice when needed. The advantage of having the mobile app installed into the mobile phones would be the convenience of access at anytime and anywhere. Our app was designed to be used off line and with pharmacological information of the medications. It is less cumbersome than carrying handwritten notes or text books. These are attributes are helpful and have been shown to assist with learning and revision [25–27]. This app has the potential to be used to teach other HCPs and even as a tool to educate patients.

Interestingly, the mobile app group did better in terms of technique with the Turbuhaler, Respimat and metered-dose devices, however, only the Turbuhaler device was significantly better that the control group. One possible reason for the superiority of the Turbohaler device is the need for a tactile feedback of turning the inhaler until a click is heard for learning to occur. The click on the actual device is usually soft and sometimes the inhalation step starts before the medication is properly loaded. Getting this right is important as the formoterol and budesonide combination which is commonly delivered via the Turbuhaler device is now considered first line of treatment for asthma [2]. The Accuhaler, Ellipta and Breezhaler device scored better for the control group. This may be so as the steps for the Accuhaler and Ellipta devices do resemble each other and using similar movements while the Breezhaler requires a physical insertion of a tablet before the actuation of the medication.

All groups achieved the least number of correct steps in metered-dose inhaler device and the most common error was Step 4 “press canister and inhale slowly”. This finding was similar to other studies [6, 10]. This is worrisome as the metered-dose inhaler is one of the commonness form of medication delivery for obstructive airway disease. This might suggest the need to concentrate efforts to improve on these steps and allow just-in-time training and revision to the convenience of the HCP.

The strength of our study is that it was a randomized clinical trial. The app was designed specifically for the purpose of improving the knowledge and technique of inhaler devices which is part of the nursing curriculum. Guo et al. have already shown that integration of mobile technology into the nursing curricula did help improve the learning experience of the students [16]. This study has several limitations. First, this was done at one time point and immediately after an intervention. This can explain why both groups felt confident with their knowledge and perform similarly well in terms of technique. Second, in this study, the participants were only limited to nursing students. The results may be different if the app was tested on medical students, HCP already in the workforce or patients. In addition to that, the sample size was small, which also limits generalization to the larger population.

## Conclusion

It is required that HCP are proficient with inhaler technique in order to deliver effective patient teaching. Using a randomised control trial, the study demonstrated the effectiveness of the app in teaching student nurses on inhaler techniques. This study also provides evidence that our mobile app designed to educate about inhalers is just as good as traditional hard copy instructions from the manufacturer but with the advantage of convenience of accessibility. Further studies are required to see if the knowledge and technique acquired is reproducible over time.

## Supplementary Information

**Additional file 1.**

## Data Availability

The datasets generated during and analyzed during the current study are not publicly available as this has not been granted by the local ethics board, but are available from the corresponding author on reasonable request.

## Abbreviations

HCP: Healthcare professionals
MDI: Metered-dose inhaler
App: Software application
MARs: Mobile app rating scale
IQR: Interquartile range
SD: Standard deviation
