# Supplementary material for: Mobile device: a useful tool to teach inhaler devices to healthcare professionals
Source: BMC Med Educ. 2022 Apr 2;22:238. doi: 10.1186/s12909-022-03302-0 (PMC8976399; doi:10.1186/s12909-022-03302-0)
Supplement: Supplementary file 1 — Additional file 1. [file 12909_2022_3302_MOESM1_ESM.pdf]

Assessment Checklist

Subject No: ________

1.1 Accuhaler inhaler

Steps	Achieved	Remark
1. Slide cover down until you hear a click		
2. Slide lever until it clicks to load dose.		
3. Exhale slowly		
4. Inhale deeply and steadily		
5. Hold breath.		
6. Exhale slowly		
7. Slide cover up to close mouthpiece.		

1.2 Turbuhaler inhaler

Steps	Achieved	Remark
1. Unscrew cap by turning to the right		
2. Turn bottom grip to the right		
3. Turn bottom grip to the left		
4. Exhale slowly		
5. Inhale deeply and steadily.		
6. Hold breath		
7. Exhale slowly		
8. Close cap		

1.3 Respimat inhaler

Steps	Achieved	Remark
1. Turn clear base in direction of the arrows		
2. Flip cap open.		
3. Exhale slowly.		
4. Inhale slowly and press on dose release button		
5. Hold breath.		
6. Exhale slowly		
7. Close cap.		

1.4 Breezhaler inhaler

Steps	Achieved	Remark
1. Remove cap.		
2. Open the mouthpiece		
3. Place capsule in chamber		
4. Close mouthpiece		
5. Press both piercing at the side once, simultaneously		
6. Exhale slowly		
7. Inhale deeply and steadily so that capsule vibrates with a whirring sound		
8. Hold breath.		
9. Exhale slowly		
10. Close cap		

1.5 Ellipta inhaler

Steps	Achieved	Remark
1. Slide cover down		
2. Exhale slowly		
3. Inhale deeply and steadily		
4. Hold breath.		
5. Exhale slowly		
6. Slide cover up to close mouthpiece		

1.6 MDI inhaler

Steps	Achieved	Remark
1. Open cap		
2. Shake inhaler		
3. Exhale slowly		
4. Press canister and inhale slowly		
5. Hold breath.		
6. Exhale slowly		
7. Close cap		
